# Health and motivation as mediators of the effects of job demands, job control, job support, and role conflicts at work and home on sickness presenteeism and absenteeism

**DOI:** 10.1007/s00420-020-01591-w

**Published:** 2020-10-24

**Authors:** G. Aronsson, J. Hagberg, C. Björklund, E. Aboagye, S. Marklund, C. Leineweber, G. Bergström

**Affiliations:** 1 Department of Psychology, Stockholm University, Stockholm, Sweden; 2 Unit of Intervention and Implementation Research for Worker Health, Institute of Environmental Medicine, Karolinska Institutet, Stockholm, Sweden; 3 Department of Clinical Neuroscience, Division of Insurance Medicine, Karolinska Institutet, Stockholm, Sweden; 4 Stress Research Institute, Stockholm University, Stockholm, Sweden; 5 Department of Occupational Health Sciences and Psychology, Centre for Musculoskeletal Research, University of Gävle, Gävle, Sweden

**Keywords:** Sickness absence, Sickness presenteeism, Mediation, Job resources, Job demands, Job support

## Abstract

**Purpose:**

The first objective was to contribute to a better understanding of the contrasting and paradoxical results in studies of work environment factors and sickness presence and sickness absence. A second objective was to examine if, and under what conditions, employees choose to replace sickness absence with sickness presence, i.e., so-called substitution.

**Methods:**

The study utilizes a large body of cross-sectional questionnaire data (*n* = 130,161) gathered in Sweden from 2002 to 2007 in connection with a comprehensive health promotion initiative. Health and motivation were analyzed as mediators of the effects of five job factors, job control, job support, job demand, role conflict and “work to family conflict” on sickness presence and absence.

**Results:**

The results concerning job demands indicate substitution in that increased job demands are associated with increased presenteeism and reduced absenteeism. The direct effect of higher job support was increased absenteeism, but via the health and motivation paths, the total effect of more social support was health-promoting and associated with a reduction in sickness absence and sickness presence. High job control emerged as the most pronounced health-promoting factor, reducing sickness presenteeism as well as absenteeism. More role conflicts and work-to-family conflicts were directly and indirectly associated with decreased health and increased absenteeism as well as presenteeism. earlier research.

**Conclusion:**

The mediation analyzes shed light on some of the paradoxes in research on sickness presenteeism and sickness absenteeism, especially regarding job demands and job support. The substitution effect is important for workplace policy and occupational health practice.

## Introduction

The main reason for sickness absence is reduced ability to work due to illness. Although sickness absence may sometimes be the only possible option, the choice to engage in sickness absenteeism, or its alternative, sickness presenteeism, is not always just a health-related decision, as factors other than health may influence whether one stays home or goes to work while ill (Aronsson and Gustafsson 2005). There seem to be mediating paths from psychosocial work factors to both sickness presence and absence behavior. In addition to health status, the choice between sickness absenteeism and presenteeism seem to be influenced by individual as well as and institutional factors (Miraglia and Johns 2016). Individual factors reflect concerns over consequences in the micro context while institutional factors relate to the organizational context and such aspects as rewards, and attendance pressure from the organization through threat of disciplinary actions, influence on sick pay, and other forms of pressure. This study is focused on psychosocial work environment factors and individual aspect that act as mediators in relation to sickness absence and sickness presence.

Considering that individuals make a choice between sickness absence and sickness absence, there is a point in analyzing these two behaviors together, but few studies have used sickness absence and sickness presence in the same model or analyses. However, sickness absenteeism and sickness presenteeism measures complete each other and form a stronger indicator of an individual’s health status than sickness absence alone (Aronsson et al. 2011; Caverley et al. 2007). The same work factors that can lead to sickness absence can lead to sickness presence depending on context and individual aspects. In cases that preclude sickness absence and staying home when ill, absenteeism may be substituted with presenteeism (Aronsson et al. 2011; Caverley et al. 2007; Pohling et al. 2016). Several researchers conclude that a more holistic research model is needed where presenteeism and absenteeism are integrated (Caverley et al. 2007; Gosselin 2018; Johns 2011; Miraglia and Johns 2016). However, there are few such studies. Two competing hypotheses concerning the relation between sickness presence and sickness absence exist (Gosselin 2018, p 131). The complementary effect suggests that these behaviors are positively connected while the substitution effect suggests a negative connection, i.e., adopting one of those behaviors presupposes that the other will not be engaged in.

The most comprehensive empirical study on mediating factors and paths between the work environment and presenteeism and absenteeism is a review and a meta-analysis of 109 samples performed by Miraglia and Johns (2016). The Job demands-resources model (Bakker and Demerouti 2007) and the demand-control-support model (Karasek and Theorell 1990) were used as the conceptual frameworks for selection of analyzed studies. The review as well as a qualitative mediation analysis by Baker-McClearn and colleagues (2010) have highlighted some of the paradoxical results found in research on sickness presenteeism, especially that both attractive (e.g., job control) and less attractive job characteristics (e.g., time pressure, lack of job support) were found to positively correlated to sickness presence.

In the meta-analytic study by Miraglia and Johns (2016) health and job attitudes (job satisfaction and motivation) were used as mediators. Their study indicates that high demands in form of challenging tasks may raise work engagement and motivate employees to spend more time working and work more intensively. This suggests that high job demands may increase sickness presence and reduce sickness absence. Strongly motivated employees go to work even when sick despite high demands, i.e., sickness presence substitutes sickness absence, which may be seen as a paradoxical result.

As work-to-family conflict was found to be an unhealthy aspect of high job demands, such conflict may be expected to negatively impact motivation and in turn increase both presenteeism and absenteeism (Miraglia and Johns 2016).

Also, in studies of job control, paradoxical results have emerged (Miraglia and Johns 2016). Job control as a resource reduces the workload or lessening pressures, which has a positive effect on health and well-being and reduces the need for sickness presence and sickness absence. But also opposite results have appeared. Having control over one’s job is associated with high motivation, which was found to stimulate sickness presenteeism, thus explaining why those with high job control decide against staying home when ill (Gerich 2019). It seems that under certain circumstances the freedom given by high job control is not used because of high job motivation.

Collegial and supervisor support were found to be related to sickness presenteeism in several ways. Supportive environments buffer job strain and poor health and increase the likelihood of being replaced in case of sickness, which reduce the need for sickness presenteeism (Miraglia and Johns 2016). Having a trusting relationship with one’s supervisor and supportive colleagues may reduce employees feeling that their sickness absence is unjustified. This would lower the incidence of sickness presenteeism and open for a positive relationship between job support and sickness absenteeism. This interpretation means that job support operates as a job resource (MacGregor et al. 2008).

However, individuals who can benefit from job support also report higher job satisfaction and motivation, which may motivate going to work despite being ill and result in a positive correlation with sickness presenteeism—good social relations, could create pressure for attendance. This may explain why job support has been found to be negatively as well as positively related to presenteeism. From this perspective, job support operates more as a job demand than a job resource (MacGregor et al. 2008).

## Aims

Against this background, and previous research especially by Miraglia and Johns (2016), a first objective of this study was to contribute to an increased knowledge on mediation and especially of mechanisms behind the differing and contrasting results in studies on sickness presenteeism. For this purpose, mediation analyses were conducted on the effect of job control, job support, job demand, role conflict and “work to family conflict” on sickness behavior using health and motivation as mediators. A second objective was to examine if, and under what conditions, employees choose to replace sickness absence with sickness presence, i.e., so-called substitution.

## Methods

The present study is cross-sectional and based on register data from AFA Insurance in Stockholm, Sweden. The register consists of questionnaire data on health and the psychosocial work environment collected among employees from the public and private sector in Sweden, including employees of private businesses, municipalities, and county councils in different parts of Sweden. It was administered in connection with the employees participating in a project (from 2002 to 2007) aimed at improving workers’ health. This initiative was not primarily a research project but it was based on valid measurement instruments and used methods developed and evaluated in former research projects (Bergstrom et al. 2008; Vingard et al. 2005). Because different organizations were involved in the study, the response rate varied. The response rates were between 65 and 94% (mean 78%) and are described in an earlier report on this occupational health initiative (Järvholm et al. 2008). Furthermore, in some organizations the questionnaire was modified and tailored to different occupational groups by removing or adding items. The survey was administered by regular mail to the employees with two reminders.

### Participants

To be included in the analyses the following criteria had to be met: (1) having been at the current workplace for at least 1 year, (2) between 18 and 65 years of age, and (3) have a complete set of data on presenteeism and sickness absenteeism available. The database originally consisted of 193,640 employees but after database clearing for research purposes and applying the mentioned criteria, 46,069 subjects were excluded (72% women, 28% men), leaving a population of 147,571 individuals. There was no information on the actual number of employees that were invited to respond to the survey, the only information available was the response rates given above. Because the main analyses used listwise deletion, 17,410 subjects with missing data for any of the variables included in the analyses were also excluded, giving a final study population of 130,161 employees, of which 73% were women and 27% men. The study population is described in Table 1. The majority was employed for municipalities, followed by county councils. The most commonly reported workplaces were schools (municipalities), hospitals (county councils), and post offices (state-owned). This information should be treated tentatively, however, since information about subject’s workplaces was missing for 45% of the population. As can be seen in the table, 12% of the study group reported being sickness present on more than 5 occasions over the previous year, and 8% reported more than 25 days on sick leave during this period. Among the 17,410 excluded employees, 63% were woman and 37% men; the mean age of this group was 47.0 years (sd 10.8) and the extent of presenteeism and absenteeism was almost identical to the study group.

**Table 1:** Descriptive data for the study population

Study group *n* = 130,161	
Gender, *n* (%)	
Women	95,640 (73)
Men	34,521 (27)
Age, m (SD)	46.8 (10.6)
Education, *n* (%)	
Compulsory school	13,078 (18)
High school	31,781 (44)
University	27,402 (38)
Employer, *n*%	
Private or state-owned company	22,616 (17)
Municipality	73,602 (57)
County council	33,943 (26)
Sickness presenteeism during the previous year, *n*%	
None	43,140 (33)
1 time	25,241 (19)
2–5 times	46,546 (36)
> 5 times	15,234 (12)
Sickness absenteeism during the previous year, *n*%	
None	40,833 (31)
1–7 days	58,022 (45)
8–24 days	20,735 (16)
25–365	10,571 (8)
Work-to-family conflict^1^, *n*%	
Very seldom or never	45,116 (35)
Rather seldom	31,729 (24)
Sometimes	38,111 (29)
Quite often	12,398 (10)
Very often or always	2807 (2)
Job demands 1–5^1^, mean (sd)	2.96 (.70)
Job control 1–5^1^, mean (sd)	2.88 (.80)
Job support 1–5^1^, mean (sd)	3.80 (.80)
Role conflict 1–5^1^, mean (sd)	2.37 (.84)
WTFC 1–5^1^, mean (sd)	2.20 (1.08)
General health, 0–100^2^, mean (sd)	86.94 (14.01)
Job motivation, 1–5^3^, mean (sd)	4.23 (.75)

^1^QPSNordic (Dallner et al. 2000)

^2^Health−related quality of Life, EQ−5D (Bjork and Norinder 1999)

^3^Job motivation (Björklund et al. 2013)

### Measurement instruments

The psychosocial workplace factors were assessed using the General Nordic Questionnaire for Psychological and Social Factors at Work (QPSNordic) if no other means of assessment is given (Dallner et al. 2000). The variables included in the questionnaire were primarily chosen based on the theoretical assumptions described in the introduction and on the results from the meta-analysis of Miraglia and Johns (2016). Furthermore, in line with this meta-analysis, global variables were preferred when possible, that is, different aspects of a construct were merged into one single index (see below). All QPS-scales have five scale steps from 1 to 5.

For the background factors of gender, age, education, and employer, we used single items (Bergstrom et al. 2008).

A global *job control* index (8 items, QPSNordic) was constructed based on all but one of the items in the control over decisions index (4 items; e.g., “Can you influence the amount of work assigned to you?”) and the control over work pace index (4 items; e.g., “Can you set your own work pace?”). The item “Can you decide when to be in contact with clients?” from the control over decisions index was omitted, because many of the respondents did not work with clients. Cronbach’s alpha was 0.82 for this index.

A global index of *job demands* (7 items, QPSNordic) was calculated based on 4 items measuring quantitative job demands (e.g., “Do you have to work overtime?”) and 3 items assessing decision demands (e.g., “Does your work require quick decisions?”). Cronbach’s alpha was 0.80.

Furthermore, a global index of *job support* (5 items, QPSNordic) at work was constructed based on indices on support from colleagues (2 items; e.g., “If needed, can you get support and help with your work from your coworkers?”) and on support from supervisor (3 items; e.g., “If needed, is your immediate superior willing to listen to your work-related problems?”). Cronbach’s alpha was low (0.61) and considered to be acceptable.

*Role conflicts* (QPSNordic) were assessed using 3 items (e.g., “Do you have to do things that you feel should be done differently?). Cronbach’s alpha was 0.72.

*Work-to-family conflict* (QPSNordic) was assessed with the item, “Do the demands of your work interfere with your home and family life?”.

*Work motivation* was measured with a modified work motivation scale developed by Björklund (Björklund et al. 2013). The questions included were the following: “Do you feel stimulated by your work tasks?”, “Are you motivated to work?”, “How often do you feel a strong will to work?” and “Would you spend less time at work if possible?” The response format ranged from “1 = never” to “5 = always”. Cronbach’s alpha was 0.78.

*Sickness presenteeism* was measured using the following item: “Has it happened over the previous 12 months that you have gone to work despite feeling that you should have taken sick leave due to your state of health?” The response options were “1 = no, never,” “2 = yes, once,” “3 = yes, 2–5 times,” and “4 = yes, more than five times” (Aronsson et al. 2000).

*Sickness absenteeism* was measured using a question from the Work Ability Index (Ilmarinen 2007). The question used was “How many days in total have you been away from work due to your own illness (sick leave, health care, treatment or examination)?” The response options were “no days,” “1–7 days,” “8–24 days,” “25–99 days,” and “100–365 days.” The categories “25–99 days” and “100–365 days” were merged for our analyses.

*General health. Health-related quality of life* (*HRQoL*) was assessed using the Swedish version of the EuroQol EQ-5D-3L (Bjork and Norinder 1999; Brooks 1996). The EQ-5D is a generic HRQoL questionnaire based on five dimensions: mobility, self-care, usual activities, pain/discomfort, and anxiety/depression. These dimensions can be used to generate individual health profiles that represent health states. In this study, we applied utility weights of health states from a general population using the Danish time-trade-off values to convert the health profiles to values between 0 and 1 (Wittrup-Jensen et al. 2009). A value of 0.00 indicates the worst possible health state while a value of 1.00 indicates the best.

### Statistical analyses

The variables were first checked for their statistical distribution and then correlational analyses were conducted using Pearson’s r. Secondly, we estimated the different parameters of the path model with manifest indicators, and their direct and indirect effects on sickness absence and sickness presence. Structural equation models (SEM) and the data program AMOS 25 was used. To assess model fit, we used different indicators. These were the root mean square residual (RMR), which is the square root of the discrepancy between the sample covariance matrix and the implied covariance matrix. Values close to 0 represent a good fit and, as a rule of thumb, values < 0.08 are deemed to be acceptable (Hooper et al. 2008). We also used the goodness of fit index (GFI). The GFI can be compared to R-squared, and ranges from 0 to 1 and is considered satisfactory when > 0.90. Furthermore, we used the adjusted goodness of fit (AGFI) acceptable value > 0.90, the comparative fit index (CFI) acceptable value > 0.95 and the Root mean square error of approximation (RMSEA), acceptable value < 0.10. We also calculated a Bayesian posterior predictive p-value, which should be near 0.5 for a correct model, with values toward the extremes of 0 or 1 indicating that a model is not plausible.

Since some of our variables were categorical and/or skewed, we decided to use the asymptotic distribution-free (ADF) method (Jones and Waller 2015). Moreover, to handle skewness resulting from the indirect effects (mediating effects) produced by the direct effects and their distributions, we used 5000 bootstrap replicates to estimate bias-corrected confidence intervals and p-values (Lockwood CM and DP. 1998; Valente et al. 2016). Finally, to check our estimates, we ran Bayesian estimation via the Markov chain Monte Carlo (MCMC) algorithm with a uniform prior (Byrne 2010; Kaplan and Depaoli 2012). The estimates of the two methods were close and in general identical when rounded to the second or third decimal place. In the sequel, we report the estimates from the ADF bootstrap procedure if not stated differently. Listwise deletion of data was used, since Amos cannot accept missing data when using any estimation criterion, such as ADF, except for when using maximum likelihood. In the results section, we will use the concept of “effect” in a statistical sense; that is, we do not imply causal effects between variables.

## Results

### Correlation patterns

Table 2 shows the correlations (Pearson) between variables in the model. As could be expected, sickness absence and sickness presence were found to be positively correlated (0.31). The mediating variables—health and motivation—negatively correlated with sickness presenteeism and with sickness absenteeism in both the correlation (Table 2) and mediation analyses (Table 4); that is, the better the health and the higher the motivation, the less presenteeism and the less absenteeism. All work factors and the two mediating variables showed relatively higher correlations with sickness presence than with sickness absence. The associations between age and sickness presence and age and sickness absence are negative, which indicates that sickness presenteeism as well as sickness absence decrease with increasing age.

**Table 2:** Correlation matrix (Pearson) for variables in the model, *n* = 130,161

	SP	SA	Job Motivation	General health	Job control	Job support	Job Demands	Role conflicts	WTFC	Sex^1^	Age
Sickness presence (SP)											
Sickness absence (SA)	.31										
Job motivation	− .21	− .15									
General health	− .37	− .30	.21								
Job control	− .18	− .13	.30	.15							
Job support	− .18	− .07	.33	.16	.32						
Job demands	.20	− .00	.03	− .08	− .16	− .14					
Role conflicts	.25	.06	− .19	− .14	− .22	− .33	.51				
WTFC	.30	.10	− .22	− .22	− .19	− .25	.47	.42			
Gender	− .05	− .10	− .13	.08	.09	− .08	− .04	.01	− .07		
Age	− .09	− .06	.20	− .08	.10	− .03	− .01	− .07	− .07	− .04	

*WTFC* Work-to-family conflict, *SP* Sickness presenteeism, *SA* Sickness absenteeism, Gender: *0* Women, *1* Men

Pearson’s *r* > 0.0054 or <  − 0.0054 are statistically significant (*p* < 0.05)

### SEM model

Fit indices for the SEM model are presented in Table 3. The AGFI, CFI, and RMSEA indicated a poor fit, whereas the RMR and GFI were acceptable according to the indices. The generally poor fit may partially be explained by the relatively weak correlations given in Table 2 (Hooper et al. 2008). However, to investigate the model fit further we dissembled the model to separate smaller submodels to identify possible problem areas. From this we discovered that the variable job demands, and its low correlation with several of the other variables, was causing convergence problems in the sub models. When it was removed the models fitted well and all coefficients were significant at the 0.05 level. However, as the overall model converged and to be true to the theoretical model described, we decided to keep all variables in the final model.

**Table 3:** Goodness of fit values

	RMR	GFI	AGFI	CFI	RMSEA
Values	.060	.975	.730	.819	.151
Rules of thumb	< .08	> 0.95	> 0.90	> .95	< .10

*RMR* Root mean square residual, *GFI* Goodness of fit, *AGFI* Adjusted goodness of fit, *CFI* Comparative fit index, *RMSEA* Root mean square error of approximation

### Mediation, presenteeism, absenteeism, and substitution

**Fig. 1:**
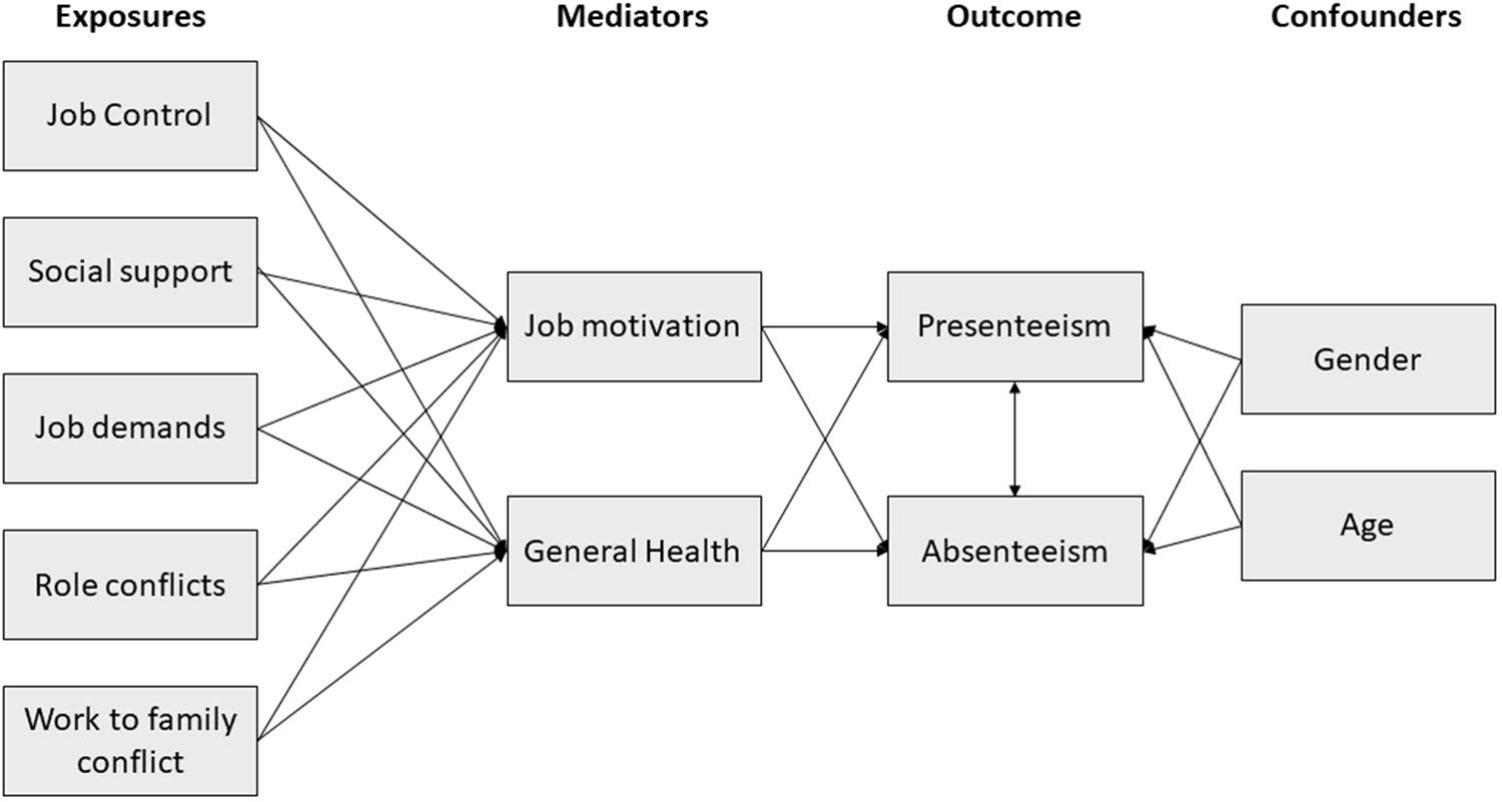
Simplified description of the analytic model showing its indirect paths. See also Table 4 for a complete description of total, direct and indirect effects

Figure 1 shows a simplified description of the analytic model depicting its indirect paths. Table 4 shows all direct, indirect and total standardised effects on presenteeism and absenteeism. Not unexpectedly, health has by far the strongest effect on sickness presence and sickness absence. In the following we use the word reduce instead of “negatively associated” etc. without indicating causality.

**Table 4:** Structural parameter estimates for direct, indirect and total effects of variables on presenteeism and absenteeism

Variable	Presenteeism	Absenteeism
Direct effect		
Job control	− .039	− .058
Job support	− .023	.013
Job demands	.076	-.049
Role conflict	.082	.012
Work-to-family conflict	.131	.027
General health	− .344	− .306
Motivation	− .092	− .082
Sex	− .021	− .085
Age	− .094	− .066
Indirect effect via motivation		
Job control	− .016	− .014
Job support	− .019	− .017
Job demands	− .019	− .017
Role conflict	.009	.008
Work-to-family conflict	.018	.016
Indirect effect via general health		
Job control	− .029	− .025
Job support	− .024	− .021
Job demands	− .017	− .015
Role conflict	.012	.011
Work-to-family conflict	.061	.054
Total indirect effects		
Job control	− .044	− .038
Job support	− .043	− .038
Job demands	− .036	− .032
Role conflict	.021	.019
Work to family conflict	.079	.070
Total effects		
Job control	− .084	− .097
Job support	− .066	− .025
Job demands	.040	− .081
Role conflict	.103	.031
Work-to-family conflict	.210	.097

All coefficients are standardised and significant at *p* level .000

#### Job control

Job control was found to reduce presenteeism directly as well as indirectly via both the health and motivation paths. Job control had the highest total reduction effect on presenteeism of all the factors (− 0.084). Job control reduced absenteeism directly (− 0.058) as well as indirectly through the health path (− 0.025) and through the motivation path (− 0.014). Among all the factors, job control has the highest total reduction effect on absenteeism (− 0.097).

#### Job support

The resource factor of job support was found to reduce presenteeism directly as well as indirectly via both the health and motivation paths. The total effect was − 0.066. The direct effect of job support on absenteeism indicates that support increased absenteeism (0.013), while the total indirect effect indicates a reduction in absenteeism (− 0.038). The total effect of job support still decreased absenteeism (− 0.025), because the reduction from the indirect effects is stronger than the direct effect.

#### Job demands

For job demands, the direct effect was found to be positive, suggesting that high job demands stimulate presenteeism (0.076). The indirect effects were in the other direction—reducing presenteeism. The total indirect effects reduced presenteeism (− 0.036), which indicates that the total effect of job demands on sickness presence is positive (0.040), because the direct positive effect was stronger than the negative indirect effects.

Job demands were found to reduce absenteeism directly (− 0.049) and indirectly through the motivation path (− 0.017) and the health path (− 0.015). The total reduction effect was − 0.081. The result indicates substitution in that the total effects of increased job demands are associated with increased presenteeism (0.040) and reduced absenteeism (− 0.081).

#### Role conflicts

Role conflict and work-to-family conflict were found to increase presenteeism directly via both paths, indicating the higher the role conflict, the higher the presenteeism. The effect of work-to-family conflict via health was strongest among all the indirect effects (0.061), and the total effect of work-to-family conflict on presenteeism was the highest of all factors (0.210).

Role conflict and work-to-family conflict were found to increase absenteeism through direct as well as through indirect effects. The total effect of work-to-family conflict on absenteeism was 0.097, which is the highest coefficient relating to absenteeism. The total effects of the two role conflict factors were stronger than those of any of the other factors.

## Discussion

Research on psychosocial work environment factors and sickness presence has shown some paradoxical results. In contrast to what might be expected, not only have high job demands in some studies been shown to be associated with good health but high job resources have also been found to be associated with ill health and sickness absence (Miraglia and Johns 2016). A reason for such findings may be that the associations between psychosocial factors and sickness presence are mediated by factors that act in opposite directions.

In a meta-analytic study, Miraglia and Johns (Miraglia and Johns 2016) identified and analyzed two pathways from the work factors, job control, job support and job demands, to sickness presence or to sickness absence. One pathway was via health and one was via job attitudes (satisfaction—motivation). In the present study, we analyzed a large body of data in relation to these two pathways and to some of the research questions raised by the meta-analytical study(Miraglia and Johns 2016). There were similarities and but also some noteworthy differences.

### Comments to mediation analyses and results

For two of the factors—job support and job demands—the mediation analysis contributed to nuanced images and increased understanding of the paradoxes in the research area.

The direct effect of job support was increased sickness absenteeism and the indirect effects were decreased sickness absence. Job support is also directly and indirectly associated with decreased presenteeism, which means that the total effects of job support is health promoting. The result may reflect that good job support encourages the kind of social climate where people do not suppress their health problems when ill. A trusting relationship with one’s supervisor and colleagues may also reduce feelings that sickness absence is unjustified. The need for sickness presence and sickness absence decrease via the health and motivation paths. In the meta-analytical study (Miraglia and Johns 2016), job support increased sickness presence via the motivation path, which probably reflected that high motivation stimulates working when ill. Why the Swedish workers act in the opposite manner is a question for further research.

The relationship for job demands is also somewhat complicated and unexpected. The direct effect of increased demands is increased presenteeism (0.076) and decreased absenteeism (− 0.049). Job demand is also connected to decreased absenteeism via the health and motivation path. The indirect effect on sickness presenteeism is a reduction in presenteeism but the effects are not so strong that they outweigh the strong direct effect in opposite direction. Paradoxically, high work demands appear to be important factor for low absenteeism. However, some of the “good” effects of high demands on sickness absence seem to be the result of substitution (total effect 0.040). Our study was cross-sectional so we cannot draw any conclusions that there are good long-term effects. Actually, it may also be that motivated employees with good health may be given, or take on the responsibility for, challenging work tasks. Previous research has shown that high sickness presence is a long term risk factor for health (Bergstrom et al. 2009) and for sickness absence (Bergström et al. 2009).

The result in our study was the reversed to the association in the meta-analytic study, where high demand was associated with high absenteeism. We can only speculate about the reason for this. One reason could be different samples. To a large extent the Swedish sample consisted of white-collar workers (60 percent had university or high school education). Increased demand in these groups may mean more of positive stimulation than of harmful load.

There may also be methodological reasons for the contrasting results. A general challenge with interpreting results on job demands concerns its potential non-linearity. A moderate increase in job demands from a low or very low level may be considered to be a health-promoting and motivating characteristic of work, provided that job resources are present, whereas an increase in job demands among employees who already work under higher demands tends to be health eroding. If this Swedish population differs from the population investigated by Miraglia and Johns (2016) concerning the distribution of the scores of the job demand variable, there could hypothetically be such effects. However, to what extent non-linearity may operate in this way and influence the results with respect to our rather heterogeneous study data is impossible to know. The effect of non-linearity is a matter for further research.

For the three remaining psychosocial factors the results were mainly in accordance with what could be expected considering previous research. Job control were directly and indirectly associated with lower absenteeism and lower presenteeism. However, in the meta-analytical study, job control increased sickness presence via the motivation path, i.e., the same difference was observed as for job support. Role conflict and work to family conflict were associated with higher presenteeism and higher absenteeism. It is of interest that the total effect of work to family conflict on presenteeism was as high as 0.21, which is far higher than any other effect.

### Future research and limitations

The study has had the ambition to contribute to a more holistic research model by analyzing mediation and combining sickness presence and sickness absence in the analyses. One conclusion is that the individuals’ decisions between engaging in sickness absence or sickness presence, i.e., substitution should have higher priority in future research, both for theoretical and practical reasons (Cooper and Lu 2018). There is also a need to develop valid measures that combine sickness presence and sickness absence (Aronsson et al. 2011; Caverley et al. 2007).

In the current study the effects of job demands were unexpected and differed from the cited meta-analysis (Miraglia and Johns 2016). A possible reason for may be the aforementioned non-linearity. The differing results may also reflect that there are different types of job demands. In occupational research, demands may be divided into quantitative and qualitative demands as well as into physical, emotional, and cognitive demands. In our study, the demand scale measured quantitative demands and demands for attentiveness and for quick or difficult decision-making. Interesting in this context is a recent meta-analytical study of the reasons for going on disability pension, which can be seen as an endpoint of sickness absence (Knardahl et al. 2017). That study found very limited evidence for an effect of job demands on disability pension. This suggests that there is a need for more specific knowledge on the relations between different types of job demands and health, motivation, sickness absence, and sickness presence. A more differentiated job demand measure is a recommendation for further research and stratified analyses related to skill demands.

Motivation as mediator seems to play somewhat different roles in our study and in the meta-analytic study (Miraglia and Johns 2016). It is difficult to understand and interpret but one possible reason may be that the corresponding indicator in the meta-analytic study was job attitudes, a factor that was composed of both motivation and job satisfaction, the latter a factor that was missing in our data.

The amount of data utilized in this study was large. It was mainly gathered from public-sector employees in Sweden, and care personnel and schoolteachers were the largest occupational groups—characteristics that deserve consideration when generalizing the results. As always, the use of cross-sectional self-reports may increase the risk for common method variance. However, for sickness presenteeism, it is difficult to find data sources other than self-reports.

A weakness is that the questionnaire was not primarily created for the purposes of conducting mediation analyses of work factors and presenteeism and some variables were questionable as indicators of comparisons with the meta-analytic study. This is something that should be considered in further research.

## Conclusions

The results concerning the total effect of job demands indicate substitution of sickness absence, that is, in case of increased job demands sickness absence tends to be replaced by sickness presence. Substitution effects are important for workplace policy and occupational health practice. In some cases, low sickness absence rate may be a false indicator of good work environment quality.

Role conflicts and work-to-family conflict were found to increase absenteeism through direct as well as through indirect effects via health and job motivation. The direct effects of job support were increased absenteeism and decreased presenteeism. However, the total effect of job support via its association with health and motivation was a reduction of sickness absence as well as of sickness presence. Job control seems to be a genuine health-promoting factor, which reduced sickness presenteeism as well as sickness absenteeism directly and indirectly. Hopefully, this study can contribute to an increased understanding of some paradoxical results in the research on sickness presence and sickness absence.

## References

[CR1] Aronsson G, Gustafsson K (2005). Sickness presenteeism: prevalence, attendance-pressure factors, and an outline of a model for research. J Occup Environ Med.

[CR2] Aronsson G, Gustafsson K, Dallner M (2000). Sick but yet at work. An empirical study of sickness presenteeism. J Epidemiol Community Health.

[CR3] Aronsson G, Gustafsson K, Mellner C (2011). Sickness presence, sickness absence, and self-reported health and symptoms. IJWHM.

[CR4] Baker-McClearn D, Greasley K, Dale J, Griffith F (2010). Absence management and presenteeism: the pressures on employees to attend work and the impact of attendance on performance. Hum Res Manag Journal.

[CR5] Bakker AB, Demerouti E (2007). The job demands-resources model: state of the art. J Manag Psycholy.

[CR6] Bergstrom G, Bjorklund C, Fried I, Lisspers J, Nathell L, Hermansson U, Helander A, Bodin L, Jensen IB (2008). A comprehensive workplace intervention and its outcome with regard to lifestyle, health and sick leave: the AHA study. Work.

[CR7] Bergstrom G, Bodin L, Hagberg J, Lindh T, Aronsson G, Josephson M (2009). Does sickness presenteeism have an impact on future general health?. Int Arch Occup Environ Health.

[CR8] Bjork S, Norinder A (1999). The weighting exercise for the Swedish version of the EuroQol. Health Econ.

[CR9] BjörklundI CJ, Lohela Karlsson M (2013). Is a change in work motivation related to a change in mental well-being?. J vocat Behav.

[CR10] Brooks R (1996). EuroQol: the current state of play. Health Policy.

[CR11] Byrne B (2010). Structural Equation modeling with AMOS, 2 edn.

[CR12] Caverley N, Cunningham JB, MacGregor JN (2007). Sickness presenteeism, sickness absenteeism, and health following restructuring in a public service organization. J Manage Stud.

[CR13] Cooper C, Lu L (2018). Presenteeism at Work.

[CR14] Dallner M, Elo A-L, Gamberale F, Hottinen V, Knardahl S, Lindström K, Skogstad A, Orhede E (2000) Validation of the General Nordic Questionnaire (QPSNordic) for Psychological and Social Factors at Work. Nord 2000:12. Nordic Council of Ministers, Copenhagen

[CR15] Gerich J (2019). Sickness presenteeism as coping behaviour under conditions of high job control. German J Hum Res Manag J.

[CR16] Gosselin E, Cooper C, Lu L (2018). The Dynamic of Assiduity at Work. Presenteeism at Work.

[CR17] Hooper D, Coughlan J, MR. M (2008). Structural equation modelling: guidelines for determining model fit. Electron J Bus Res Methods.

[CR18] Ilmarinen J (2007). The work ability index. Occup Med.

[CR19] Johns G (2011). Attendance dynamics at work: the antecedents and correlates of presenteeism, absenteeism, and productivity loss. J Occup Health Psych.

[CR20] Jones JA, Waller NG (2015). The normal-theory and asymptotic distribution-free (ADF) covariance matrix of standardized regression coefficients: theoretical extensions and finite sample behavior. Psychometrika.

[CR21] Järvholm B, Reuterwall C, Waling K (2008) Evaluation of AFAs Work Environment Support to Municipalities and County Councils (In Swedish). Stockholm. https://www.suntliv.nu/AFATemplates/Page.aspx?id=11541

[CR22] Kaplan D, Depaoli S (2012). Bayesian structural equation modeling.

[CR23] Karasek R Theorell T (1990) Healthy work. Stress, productivity and the reconstruction of working life. Basic Books, New York

[CR24] Knardahl S, Johannessen HA, Sterud T, Harma M, Rugulies R, Seitsamo J, Borg V (2017). The contribution from psychological, social, and organizational work factors to risk of disability retirement: a systematic review with meta-analyses. BMC Public Health.

[CR25] Lockwood CM, DP. M (1998) Bootstrapping the standard error of the mediated effect. Paper presented at the The Twenty-Third Annual SAS Users Group International Conference, Nashville, Tennessee.

[CR26] MacGregor JN, Barton Cunningham J, Caverley N (2008). Factors in absenteeism and presenteeism: life events and health events. Manag Res News.

[CR27] Miraglia M, Johns G (2016). Going to work ill: a meta-analysis of the correlates of presenteeism and a dual-path model. J Occup Health Psychol.

[CR28] Pohling R, Buruck G, Jungbauer KL, Leiter MP (2016). Work-related factors of presenteeism: the mediating role of mental and physical health. J Occup Health Psychol.

[CR29] Valente MJ, Gonzalez O, Miocevic M, MacKinnon DP (2016). A note on testing mediated effects in structural equation models: reconciling past and current research on the performance of the test of joint significance. Educ Psychol Meas.

[CR30] Vingard E, Lindberg P, Josephson M, Voss M, Heijbel B, Alfredsson L, Stark S, Nygren A (2005). Long-term sick-listing among women in the public sector and its associations with age, social situation, lifestyle, and work factors: a three-year follow-up study. Scand J Public Health.

[CR31] Wittrup-Jensen KU, Lauridsen J, Gudex C, Pedersen KM (2009). Generation of a Danish TTO value set for EQ-5D health states. Scand J Public Health.

